# Female researchers in high-impact psychiatric journals: What do they focus on?

**DOI:** 10.3389/fpsyt.2023.1104683

**Published:** 2023-02-14

**Authors:** Melanie Trimmel, Michaela Amering, Stefanie Suessenbacher-Kessler, Beate Schrank, Andrea Gmeiner

**Affiliations:** ^1^Clinical Division of Social Psychiatry, Department of Psychiatry and Psychotherapy, Medical University of Vienna, Vienna, Austria; ^2^Department of Adult Psychiatry, Karl Landsteiner University for Health Sciences, University Clinic Tulln, Tulln, Austria

**Keywords:** academic psychiatry, gender, bibliometric analysis, medical literature, psychiatry research, women in medicine

## Abstract

The regular analysis of publication trends, including gender aspects, is an important contribution to the identification of gender-specific differences in academic psychiatry. The present study aimed to characterise publication topics in three high-impact psychiatric journals across three time points within 15 years (2004, 2014 and 2019). Publication patterns of female authors compared to their male colleagues were examined. All articles published in 2019 in the three high-impact psychiatry journals JAMA Psychiatry, British Journal of Psychiatry and American Journal of Psychiatry were included and compared with data from the 2004 and 2014 assessments. Descriptive statistics were calculated and Chi-square tests were performed. In 2019, a total of 473 articles were published, 49.5% were original research articles, of which 50.4% were published by female first authors. The results of this study showed a stable trend in the publication of research on mood disorders as well as schizophrenia and psychotic disorders in high-ranking psychiatric journals. Although the percentage of female first authors in the three most common target populations under study (mood disorders, schizophrenia and general mental health) increased from 2004 to 2019, gender equality has not yet been achieved in these fields. However, in the two most frequent subject matters, basic biological research and psychosocial epidemiology, the percentage of female first authors was more than 50%. Consistent monitoring of publication trends and gender distribution by researchers and journals in psychiatric research should be continued to identify and counteract the possibility of the underrepresentation of women in certain fields.

## 1. Introduction

Bibliometric researchers outline publication trends, changes over time and specific research interests through the characterisation, the systematic assessment and the analysis of medical literature (1–10). The growing contribution of women in academic psychiatry is an area of current interest. Notably, females are strongly represented amongst both medical students, e.g. 50.5% in the United States in 2019 (11), and psychiatric residents, e.g. 48.9% in the United States in 2019 (12). Scientific publishing as one of the most important metrics of academic productivity is one criterion in hiring processes for leading positions and plays a key role in achieving higher academic ranks (13, 14).

Two recent studies demonstrated that the percentage of female authors is on the rise and gender parity in first authorship was finally achieved in the category of original research articles in 2019 (14, 15). However, women remain underrepresented in senior and leadership positions, and in academic medicine (14–19). This fact bears the risk that issues relevant to women are systematically underrepresented, thus potentially resulting in a loss of diversity with gaps in psychiatric research (20, 21).

As a result, there has been a call for specific interventions to address gender inequalities in academic psychiatry and to tackle potential barriers to women’s career advancements (17, 22–24). The ongoing analysis of publication trends, including gender aspects, provides an important contribution to the identification of potential gender-specific differences in medical research (25–27). The present study aimed to characterise psychiatric research in three high-impact psychiatric journals in order to explore changes in publication topics across three time points within 15 years (2004, 2014 and 2019). Furthermore, publication patterns of women compared to their male colleagues were analysed.

## 2. Materials and methods

A bibliometric review of all articles published in 2019 in the three high-impact psychiatry journals JAMA Psychiatry, British Journal of Psychiatry and American Journal of Psychiatry was conducted in order to explore subject matters, subspecialty areas and target populations under study, considering gender distribution in authorship. A comparison with data from the 2004 and 2014 assessments of subspecialty areas and target populations under study was made. The first similar assessment by the study group was conducted in 1994. Back in 1994, the highest-ranking general psychiatry journals (Archives of General Psychiatry (now: JAMA Psychiatry) and The American Journal of Psychiatry) as well as the highest-ranking non-American journal (The British Journal of Psychiatry) were selected due to their longstanding consistency in high-impact factor ranks. To ensure comparability with previous years, these journals were then kept for follow-up examinations. Articles stated as published in-print on the journals’ homepages were included. A distinction was made between original research articles as defined by the journals and non-original research articles (e.g. editorials, commentaries, letters). One month’s worth of data from each journal were assessed independently by two researchers to evaluate the inter-rater reliability. The remaining 11 months’ data were assessed by two researchers together. Gender was identified by the author’s gender-specific given names. In cases where the gender of the author could not be clearly identified by the given name, university homepages or research gate profiles were screened for further information. Gender was determined for all authors indicating first or senior authorship with a single authorship counted as both first and senior authors.

The authors developed and reconciled the following coding scheme based on a previous study by this study group (25). For all original research articles in 2019, the study group distinguished the subject matter as (1) interventions (either psychotherapy, psychoeducation, psychopharmacotherapy, combined/complex intervention or other somatic therapy), (2) epidemiology (either biological, psychosocial or other variables), (3) basic research (either biological or psychosocial variables), (4) clinical research, (5) development or validation of measurement scales or (6) economic evaluation. Target populations under study and subspecialty areas were also coded for all articles (original research and non-original research articles) in 2004, 2014 and 2019. Amongst subspecialty area, the main topics or special interests of the publications were classified (further details see Supplementary material). For each article, a maximum of three codes related to mental health conditions and subspecialty areas were allocated, respectively. The age group of participants under investigation was coded for all articles to distinguish amongst research about children and adolescents (until the age of 18), adults (aged 18 to 65) and the elderly (older than 65 years). The data collected in 2019 were compared to existing data from 2004 to 2014 (28). Statistical analyses were performed using SPSS (SPSS 27). Descriptive statistics (percentages and frequencies) were calculated for each category. For each year, the percentages for each category were calculated by dividing the number of target populations under study or subspecialty area by the number of all codes assigned to the category in total. For each year, percentages were calculated for each gender by dividing the number of first authors of that gender by the total number of first authors (including unclear gender). Chi-square tests were performed in order to calculate developments from 2004 to 2019 and to compare the results of female and male authors. *p* values of <0.05 were considered to be statistically significant. Inter-rater reliability was calculated in a 10% random subsample of all articles *via* Cohen’s kappa of 0.87.

## 3. Results

A total of 473 articles were published in 2019. Of those 234 (49.5%) were original research articles, of which 118 were published by females as first authors (15). In 2014, the total number of published articles was 642. 318 (49.5%) accounted for original research articles, including 133 by females as first authors. In 2004, the number of all articles was 800, with 502 (62.8%) accounting for original research articles, including 182 with female first authors (27).

### 3.1. Mental health condition and target population

**Figure 1:**
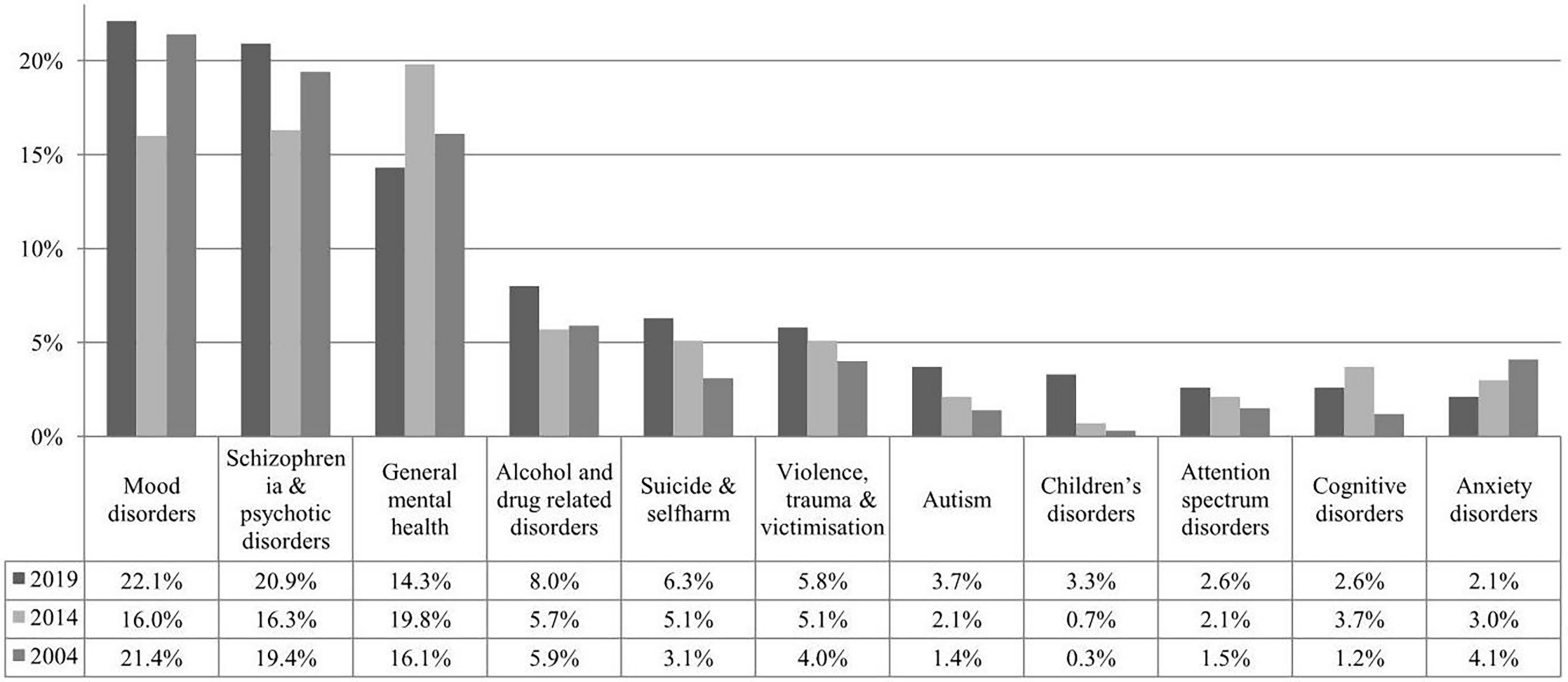
Percentages of articles by mental health condition and target population under study in 2004, 2014 and 2019. For each year, percentages were calculated for each category, by dividing the number of that condition by the number of all codes assigned to the conditions (*n* = 574 in 2019, *n* = 724 in 2014 and *n* = 948 in 2004).

In 2019, a total of 574 codes were assigned to this category for all articles. In 2014, 724 codes were allocated, and 948 codes in 2004. In 2019, the largest number of articles published focused on mood disorders (*n* = 127 of 574; 22.1%) and schizophrenia and psychotic disorders (*n* = 120 of 574; 20.9%). A similar finding was shown both in 2004 (mood disorders *n* = 203 of 948; 21.4%, schizophrenia *n* = 184 of 948; 19.4%) and 2014 (mood disorders *n* = 116 of 724; 16%, schizophrenia *n* = 118 of 724; 16.3%), although in 2014, the broader category of general mental health (three or more diagnostic categories) ranked first (*n* = 143 of 724; 19.8%). The percentage of articles on other conditions with publication numbers of 15 or more in 2019 increased on the topics of alcohol- and drug-related disorders, suicide and self-harm, violence, trauma and victimisation, autism, children’s disorders, attention spectrum disorders and cognitive disorders, whilst there was a decrease in research on anxiety disorders since 2004 (see Figure 1).

When examining articles by female first authors, a similar picture emerges. The three most common categories in all 3 years observed were mood disorders, schizophrenia and general mental health with an increase in the percentages of female first authors in these conditions from 2004 to 2019 (mood disorders 31.5 to 37.8%, schizophrenia 22.3 to 39.2%, general mental health 26.8 to 32.9%). In 2019, the percentage of female first authors was almost equivalent to male first authors for articles on alcohol- and drug-related disorders (47.8%) and autism (47.6%). Whilst articles on suicide and self-harm (52.8%), violence, trauma and victimisation (60.6%), children’s disorders (68.4%), attention spectrum disorders (60%) and anxiety disorders (58.3%) were published more frequently by female first authors. Results are also shown in Table 1.

**Table 1:** Number of female and male first authors of articles by mental health condition and target population under study in 2004, 2014 and 2019.

Mental health condition and target population under study	2019		2014		2004		
	Women *n* (%)	Men *n* (%)	Women *n* (%)	Men *n* (%)	Women *n* (%)	Men *n* (%)	Value of *p*
Mood disorders	48 (37.8)	79 (62.2)	45 (38.8)	66 (56.9)	64 (31.5)	130 (64)	0.544
Schizophrenia and psychotic disorders	47 (39.2)	73 (60.8)	30 (25.4)	84 (71.2)	41 (22.3)	131 (71.2)	0.100
General mental health	27 (32.9)	55 (67.1)	34 (23.8)	104 (72.7)	41 (26.8)	102 (66.7)	0.314
Alcohol- and drug-related disorders	22 (47.8)	24 (52.2)	20 (48.8)	19 (46.3)	24 (42.9)	28 (50)	0.731
Violence and trauma and victimisation	20 (60.6)	13 (39.4)	21 (56.8)	16 (43.2)	18 (47.4)	19 (50)	0.354
Suicide and self-harm	19 (52.8)	17 (47.2)	12 (32.4)	24 (64.9)	13 (44.9)	16 (55.2)	0.102
Children’s disorders	13 (68.4)	6 (31.6)	4 (80)	1 (20)	2 (66.7)	1 (33.3)	**<0.001**
Autism	10 (47.6)	11 (52.4)	9 (60)	6 (40)	5 (38.5)	8 (61.5)	0.094
Attention spectrum disorders	9 (60)	6 (40)	7 (46.7)	8 (53.3)	4 (28.6)	9 (64.3)	0.081
Anxiety disorders	7 (58.3)	4 (33.3)	5 (22.7)	16 (72.7)	13 (33.3)	26 (66.7)	0.341
Cognitive disorders	4 (26.7)	11 (73.3)	5 (18.5)	20 (74.1)	2 (18.2)	9 (81.8)	0.293

For each year, percentages were calculated for each gender by dividing the number of first authors of that gender by the total number of first authors (including unclear gender) for the mental health condition and target population. Chi-square tests were performed to calculate developments from 2004 to 2019. *p* values <0.05 were considered to be statistically significant.

### 3.2. Subspecialty areas

In this category, a total of 635 codes were assigned for all articles in 2019, 689 codes in 2014 and 760 in 2004. In most subspecialty areas, the proportion of topics remained relatively stable between 2004 and 2019, notable changes were observed in some areas of interest (see Table 2). The number of papers published on neuroimaging almost halved from 105 articles (of 760; 13.8%) in 2004 to 58 (of 635; 9.1%) in 2019. Whereas the percentage of female first authors of publications on neuroimaging increased considerably (from 30 (of 105; 28.6%) in 2004 to 25 (of 58; 43.1%) in 2019). In other frequently published areas, an increase in the number of papers was observed: For example, pharmacotherapy (from 81 (of 760; 10.7%) in 2004 to 93 (of 635; 14.7%) in 2019), genetics (from 46 (of 760; 6%) in 2004 to 51 (of 635; 8%) in 2019) and psychotherapy and psychological intervention (from 35 (of 760; 4.6%) in 2004 to 46 (of 635; 7.2%) in 2019).

**Table 2:** Number of articles and number of female and male first authors of articles by subspecialty area in 2004, 2014 and 2019.

Subspecialty areas	2019			2014			2004		
	Overall *n* (%)	Women *n* (%)	Men *n* (%)	Overall *n* (%)	Women *n* (%)	Men *n* (%)	Overall *n* (%)	Women *n* (%)	Men *n* (%)	Value of *p*
Pharmacotherapy	93 (14.7)	30 (32.3)	63 (67.7)	79 (11.5)	21 (26.6)	55 (69.6)	81 (10.7)	23 (28.4)	54 (66.7)	0.291
Neuroimaging	58 (9.1)	25 (43.1)	33 (56.9)	76 (11)	36 (47.4)	39 (51.3)	105 (13.8)	30 (28.6)	70 (66.7)	0.504
Other	57 (9)	23 (40.4)	34 (59.7)	38 (5.5)	11 (29)	26 (68.4)	26 (3.4)	5 (19.2)	21 (80.8)	**<0.001**
Genetics	51 (8)	29 (56.9)	22 (43.1)	32 (4.6)	13 (40.6)	18 (56.3)	46 (6)	16 (34.8)	26 (56.5)	**0.039**
Psychotherapy and Psychological intervention	46 (7.2)	22 (47.8)	23 (50)	56 (8.1)	17 (30.4)	39 (69.6)	35 (4.6)	12 (34.3)	23 (65.7)	0.071
Prevention	27 (4.3)	12 (44.4)	15 (55.6)	8 (1.2)	1 (12.5)	7 (87.5)	1 (0.1)	1 (100)	0 (0)	**0.002**
Addiction	23 (3.6)	9 (39.1)	14 (60.9)	30 (4.4)	14 (46.7)	15 (50)	36 (4.7)	23 (63.9)	11 (30.6)	**0.012**
Cognition	22 (3.5)	10 (45.5)	12 (54.6)	31 (4.5)	13 (41.9)	17 (54.8)	24 (3.2)	10 (41.7)	14 (58.3)	0.980
Research on research	21 (3.3)	11 (52.4)	10 (47.6)	5 (0.7)	1 (20)	4 (80)	4 (0.5)	2 (50)	2 (50)	**0.011**
Ethics and human rights, philosophy and concepts	21 (3.3)	6 (28.6)	15 (71.4)	10 (1.5)	0 (0)	10 (100)	27 (3.6)	6 (22.2)	20 (74.1)	0.984
Aetiology	21 (3.3)	9 (42.9)	12 (57.1)	14 (2)	7 (50)	7 (50)	7 (0.9)	1 (14.3)	6 (85.7)	**0.011**
Policy	20 (3.2)	6 (30)	14 (70)	10 (1.5)	3 (30)	7 (70)	1 (0.1)	0 (0)	1 (100)	**0.013**
Somatic illness in psychiatric patients	19 (3)	6 (31.6)	13 (68.4)	24 (3.5)	11 (45.8)	11 (45.8)	16 (2.1)	4 (25)	11 (68.8)	0.512
Woman’s (health) issues	18 (2.8)	10 (55.6)	8 (44.4)	13 (1.9)	9 (69.2)	4 (30.8)	17 (2.2)	9 (52.9)	8 (47.1)	0.796
Psychopathology, diagnostics and nomenclature	16 (2.5)	7 (43.8)	9 (56.3)	25 (3.6)	7 (28)	18 (72)	15 (2)	3 (20)	12 (80)	0.195

For each year, percentages were calculated for each category, by dividing the number of that subspecialty area by the number of all codes assigned to subspecialty areas (*n* = 635 in 2019, *n* = 689 in 2014 and *n* = 760 in 2004). For each year, percentages were calculated for each gender by dividing the number of first authors of that gender by the total number of first authors (including unclear gender) for the subspecialty area (with an annual number of at least 15 in 2019). Chi-square tests were performed to calculate developments from 2004 to 2019. *p* values <0.05 were considered to be statistically significant.

Likewise, the proportion of female first authors in these fields increased (pharmacotherapy 23 (of 81; 28.4%) in 2004 to 30 (of 93; 32.3%) in 2019; psychotherapy and psychological intervention 12 (of 35; 34.3%) in 2004 to 22 (of 46; 47.8%) in 2019), in particular the percentage of published articles on genetics by female first authors rose greatly from 16 (of 46; 34.8%) in 2004 to 29 (of 51; 56.9%) in 2019. The third most common category in 2019 ‘other’ (summary topics) grew considerably in comparison to 2004 (from 26 (of 760; 3.4%) to 57 (of 635; 9%)); similarly, the percentage of female first authors in this category increased from 19.2% (*n* = 5) in 2004 to 40.4% (*n* = 23) in 2019. Another considerable increase was found in the subspecialty areas prevention, research on research, aetiology and policy, both overall and amongst female first authors, whereas the field of addiction showed a marked decline from 2004 to 2019, both overall and in the percentage of female first authors (see Table 2).

### 3.3. Subject matters

**Figure 2:**
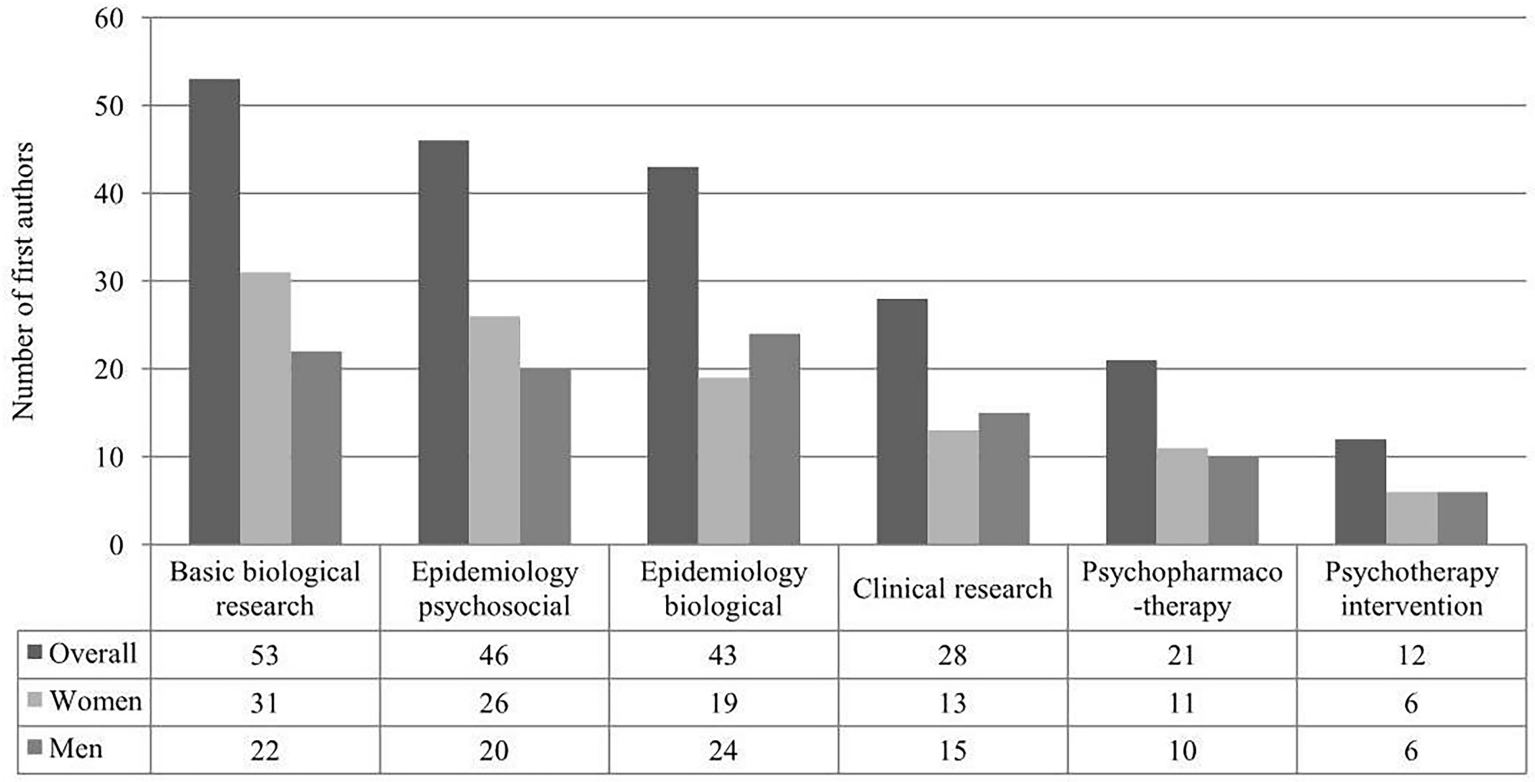
Number of female and male first authors of original research articles (*n* = 234) by subject matter (illustration of the six highest ranked) in 2019.

In 2019, the largest share amongst subject matters of original research articles (*n* = 234) was accounted for basic biological research (22.7%; *n* = 53), followed by epidemiological psychosocial (19.7%; *n* = 46), epidemiological biological (18.4%; *n* = 43), clinical research (12%; *n* = 28) and psychopharmacotherapeutic interventions (9%; *n* = 21), see Figure 2. Female first authors account for a remarkably larger percentage compared to their male colleagues in the top-ranked subject matter categories of basic biological research (58.5%; 31 of 53), epidemiological psychosocial (56.5%; 26 of 46) and pharmacotherapeutical interventions (52.4%, 11 of 21). Results are also shown in Table 3.

**Table 3:** Number of articles and number of female and male first authors of original research articles by subject matter in 2019.

Subject matter (*n* = 234)	2019			
	Overall *n* (%)	Women *n* (%)	Men *n* (%)	Value of *p*
*Intervention*				
Psychotherapy	12 (5.1)	6 (50)	6 (50)	0.976
Psychoeducation	8 (3.4)	3 (37.5)	5 (62.5)	0.457
Psychopharmacotherapy	21 (9)	11 (52.4)	10 (47.6)	0.851
Combined pharmacotherapy and psychotherapy	5 (2.1)	3 (60)	2 (40)	0.665
Other somatic therapy	3 (1.3)	0 (0)	3 (100)	0.079
*Epidemiology*				
Biological	43 (18.4)	19 (44.2)	24 (55.8)	0.365
Psychosocial	46 (19.7)	26 (56.5)	20 (43.5)	0.356
Other	10 (4.3)	4 (40)	6 (60)	0.500
*Basic research*				
Biological	53 (22.7)	31 (58.5)	22 (41.5)	0.182
Psychosocial	2 (0.9)	0 (0)	2 (100)	0.152
*Other*				
Clinical research	28 (12)	13 (46.4)	15 (53.6)	0.652
Development or validation of measurement scales	1 (0.4)	1 (100)	0 (0)	0.320
Economic evaluation	2 (0.9)	1 (50)	1 (50)	0.990

For each year, percentages were calculated for each category, by dividing the number of that subject matter by the number of all original research articles (*n* = 234). For each year, percentages were calculated for each gender by dividing the number of first authors of that gender by the total number of first authors (including unclear gender) for the subject matter. Chi-square tests were performed to compare the results of female and male authors. *p* values <0.05 were considered to be statistically significant.

### 3.4. Age groups

In 2019, almost two-thirds of articles related to the child and adolescent population were published by female first authors (27 of 41 (65.9%)). In contrast, articles on adult and geriatric populations were less frequently first authored by women (adults 74 of 161 (46%); elderly 6 of 15 (40%)).

## 4. Discussion

The present study examined how publication topics in psychiatric research in three high-impact psychiatric journals have changed over three time points within 15 years, with a focus on publication patterns of female authors.

Regarding the target population under study, our findings are in line with previous studies that have shown a stable trend in the publication of research on mood disorders and schizophrenia and psychotic disorders in high-ranking psychiatric journals (4, 25). This fact raises the question of the ratio of submitted to published papers on different mental health conditions under study (4). If certain mental health conditions were more likely to be published, this would also be an influential factor for further research trends, as research funding often seems to be linked to the researchers’ academic productivity in high-impact journals (29). Furthermore, considering the importance of adequate financial support for research and its influence on publication trends, the fact that women have significantly lower chances of receiving grants is even more serious (30). Overall, this replication of the cycle of publication in high-ranking journals leading to a greater likelihood of attracting funding for more research on specific topics could result in a barrier towards comprehensive scientific advances, particularly in specific patient groups (4). In this context, it should also be critically reflected that women still make up only a small portion of the editorial boards of psychiatric journals, with women representing 30.4% of editorial boards (31) and 10.4% of editors-in-chief (32). The question of whether the proportion of women on editorial boards influences publications in terms of gender of authors, target population under study and subspecialty area remains to be answered. However, publication patterns might be influenced by a range of factors, such as editorial policy, number of submitted manuscripts on different topics and prevailing conceptual orientations (33). The fact that women are underrepresented in editorial boards (31, 32) might also contribute to other disparities including grant funding, academic promotion and compensation, which, in turn, could prevent women from reaching their full career potential. When women leave academic medicine in the course of their careers, whether for personal reasons or due to career barriers, this can result in a loss of valuable knowledge. This, in turn, can lead to a loss of diversity and augment possible gaps in psychiatric science, which could impede the progress of scientific knowledge relevant to the treatment of patients (34).

Although this study showed that the percentage of female first authors in the three most common target populations under study (mood disorders, schizophrenia and general mental health) increased from 2004 to 2019, gender equality has not yet been achieved in these fields. In other, less frequently studied target populations under study (children’s disorders, attention spectrum disorders, violence and trauma and victimisation) the percentage of female first authorship was higher than male first authorship in more than one point in time. Correspondingly, the percentage of female first authors in articles on the child and adolescent population was considerably higher (65.9%).

Looking more closely at the subspecialty areas, neuroimaging, the second most common category in 2019, has shown a remarkable decline since 2004. Meanwhile, the percentage of female first authors on neuroimaging articles increased clearly, although this seems to be mainly due to the decline in the number of articles by male first authors. A different picture emerged for articles on genetics: whilst there was only a small increase overall, the percentage of female first authors rose consistently to over 50 %. In other top-ranked areas of interest (psychopharmacotherapy, psychotherapy and psychological intervention), the number of publications increased both overall and amongst female first authors. In the two most frequent subject matters, basic biological research and epidemiology psychosocial, the percentage of female first authors was more than 50 percent. To sum up, the top fields seem to be interesting for female researchers as well. The marked increase in the number of publications from 2004 to 2019 in the subspecialty areas of prevention, research on research, aetiology and policy as well as the decrease in the subspecialty area of addiction could be interpreted as an emerging trend in certain topics. However, the interpretation appears to be limited by the fact that this is a comparison of three points in time and thus further observation seems necessary to assess the potential trend, as these changes could also be caused by thematic focuses within the journals.

Limited to three high-impact journals, the results of this study cannot be generalised to medium- or low-impact journals. Additionally, it should be taken into account that the majority of papers were published by authors from high-income areas, mainly North America and Europe (15). An analysis of social psychiatric research from Germany showed similar results with regard to mental health conditions under study, with articles on psychiatric patients in general ranked first, followed by schizophrenia, substance-related disorders and affective disorders (35). A study of mental health research in Iran showed that the most frequently published articles were on mood disorders and substance-related disorders, whilst articles on psychotic disorders accounted for a much smaller proportion. Further, this study reported regarding the general area of research, that most articles belonged to psychology (52.6%) and clinical research (31.1%), with neuroscience far behind (14.3%) (36). Local differences in publication trends thus underline the importance of aiming for geographical diversity in international high-impact journals to ensure a balanced and comprehensive publication landscape, but there is still a lot of work to be done. So far, the leading countries regarding the number of publications and impact have been the US and the UK (5, 37). A further limitation is that the present study used a binary gender system according to given names; therefore, no statements about authors identifying as non-binary can be made.

Ongoing monitoring of publication trends and gender distribution by researchers and journals in psychiatric research should be continued to identify and counteract the possibility of the underrepresentation of women in certain fields through targeted interventions (e.g. mentoring, promotion and women’s quotas).

## Data availability statement

The original contributions presented in the study are included in the article/Supplementary materials, further inquiries can be directed to the corresponding author.

## Author contributions

AG, MT and MA: study conception and design and draft manuscript preparation. AG, MT, BS and SS-K: data collection. AG and MT analysis and interpretation of results. All authors contributed to the article and approved the submitted version.

## Funding

Open access funding provided by Medical University of Vienna.

## Conflict of interest

The authors declare that the research was conducted in the absence of any commercial or financial relationships that could be construed as a potential conflict of interest.

## Publisher’s note

All claims expressed in this article are solely those of the authors and do not necessarily represent those of their affiliated organizations, or those of the publisher, the editors and the reviewers. Any product that may be evaluated in this article, or claim that may be made by its manufacturer, is not guaranteed or endorsed by the publisher.

## Supplementary material

The Supplementary material for this article can be found online at: https://www.frontiersin.org/articles/10.3389/fpsyt.2023.1104683/full#supplementary-material

## References

[1] Stone K Whitham EA Ghaemi SN. A comparison of psychiatry and internal medicine: a bibliometric study. Acad Psychiatry. (2012) 36:129–32. doi: 10.1176/appi.ap.10030048, PMID: 22532204

[2] Hodgkins P Arnold LE Shaw M Caci H Kahle J Woods AG. A systematic review of global publication trends regarding long-term outcomes of ADHD. Front Psych. (2011) 2:84. doi: 10.3389/fpsyt.2011.00084 PMC3260478 22279437

[3] Moncrieff J Crawford MJ. British psychiatry in the 20th century--observations from a psychiatric journal. Soc Sci Med. (2001) 53:349–56. doi: 10.1016/S0277-9536(00)00338-5, PMID: 11439818

[4] Murray SB Pila E Mond JM Mitchison D Nauman E Griffiths S. Global trends in high impact psychiatry research. World Psychiatry. (2018) 17:368–70. doi: 10.1002/wps.20573, PMID: 30192099 PMC6127763

[5] Fava GA Ottolini F. International trends in psychiatric research. A citation analysis. Curr Opin Psychiatry. (2004) 17:283–7. doi: 10.1097/01.yco.0000133831.04049.32

[6] Duan L Zhu G. Mapping theme trends and knowledge structure of magnetic resonance imaging studies of schizophrenia: a Bibliometric analysis from 2004 to 2018. Front Psych. (2020) 11:27. doi: 10.3389/fpsyt.2020.00027, PMID: 32116844 PMC7019376

[7] Soares EE Thrall JN Stephens TN Rodriguez Biglieri R Consoli AJ Bunge EL. Publication trends in psychotherapy: Bibliometric analysis of the past 5 decades. Am J Psychother. (2020) 73:85–94. doi: 10.1176/appi.psychotherapy.20190045, PMID: 32506985

[8] Morlino M Lisanti F Gogliettino A de Girolamo G. Publication trends of papers on schizophrenia. A 15-year analysis of three general psychiatric journals. Br J Psychiatry. (1997) 171:452–6. doi: 10.1192/bjp.171.5.452 9463605

[9] Nuryana Z Murshidi GA Rahman A. Publication trends related to schizophrenia, mental health, and depression during COVID-19. Asian J Psychiatr. (2021) 66:102878. doi: 10.1016/j.ajp.2021.102878, PMID: 34634657 PMC8485721

[10] Strand M Bulik CM. Trends in female authorship in research papers on eating disorders: 20-year bibliometric study. BJPsych Open. (2018) 4:39–46. doi: 10.1192/bjo.2017.8, PMID: 29467058 PMC6020273

[11] Heiser S (2019). The majority of U.S. medical students are women, new data show. Available from: https://www.aamc.org/news-insights/press-releases/majority-us-medical-students-are-women-new-data-show (Accessed November 12, 2022).

[12] American Psychiatric Association (2022). 2020 resident/fellow census. Available from: https://www.psychiatry.org/residents-medical-students/medical-students/resident-fellow-census (Accessed November 12, 2022).

[13] Reed DA Enders F Lindor R McClees M Lindor KD. Gender differences in academic productivity and leadership appointments of physicians throughout academic careers. Acad Med. (2011) 86:43–7. doi: 10.1097/ACM.0b013e3181ff9ff2, PMID: 21099390

[14] Hart KL Frangou S Perlis RH. Gender trends in authorship in psychiatry journals from 2008 to 2018. Biol Psychiatry. (2019) 86:639–46. doi: 10.1016/j.biopsych.2019.02.010, PMID: 30935668 PMC6699930

[15] Gmeiner A Trimmel M Gaglia-Essletzbichler A Schrank B Süßenbacher-Kessler S Amering M. Diversity in high-impact psychiatric publishing: gender parity within reach? Arch Womens Ment Health. (2022) 25:327–33. doi: 10.1007/s00737-021-01202-8 35024945 PMC8756164

[16] Carnes M Morrissey C Geller SE. Women’s health and women’s leadership in academic medicine: hitting the same glass ceiling? J Womens Health (Larchmt). (2008) 17:1453–62. doi: 10.1089/jwh.2007.0688, PMID: 18954235 PMC2586600

[17] Doyle M Pederson A Meltzer-Brody S. Demographic and personal characteristics of male and female chairs in academic psychiatry. Acad Psychiatry. (2016) 40:402–9. doi: 10.1007/s40596-015-0408-8, PMID: 26307364

[18] Bendels MHK Wanke EM Benik S Schehadat MS Schöffel N Bauer J. The gender gap in highest quality medical research - a scientometric analysis of the representation of female authors in highest impact medical journals. Dtsch Med Wochenschr. (2018) 143:e85–94. doi: 10.1055/s-0044-102267, PMID: 29727882

[19] Dhingra S Killaspy H Dowling S. Gender equality in academic psychiatry in the UK in 2019. BJPsych bulletin. (2021) 45:153–8. doi: 10.1192/bjb.2020.116, PMID: 33228828 PMC9059301

[20] Menon V Varadharajan N Bascarane S Andrade C. Trends in female authorship in two major psychiatry journals in India. Psychiatry Res. (2022) 313:114621. doi: 10.1016/j.psychres.2022.114621, PMID: 35588555

[21] Penny M Jeffries R Grant J Davies SC. Women and academic medicine: a review of the evidence on female representation. J R Soc Med. (2014) 107:259–63. doi: 10.1177/0141076814528893, PMID: 24739380 PMC4093756

[22] Sheikh MH Chaudhary AMD Khan AS Tahir MA Yahya HA Naveed S. Influences for gender disparity in academic psychiatry in the United States. Cureus. (2018) 10:e2514. doi: 10.7759/cureus.2514, PMID: 29942717 PMC6015990

[23] Borlik MF Godoy SM Wadell PM Petrovic-Dovat L Cagande CC Hajirnis A. Women in academic psychiatry: inequities, barriers, and promising solutions. Acad Psychiatry. (2021) 45:110–9. doi: 10.1007/s40596-020-01389-5, PMID: 33532916

[24] Coverdale J Aggarwal R Morreale M Balon R Beresin EV Guerrero APS. Promoting women to editorial leadership positions at academic psychiatry. Acad Psychiatry. (2021) 45:4–6. doi: 10.1007/s40596-021-01409-y, PMID: 33544377

[25] Amering M Schrank B Sibitz I. The gender gap in high-impact psychiatry journals. Acad Med. (2011) 86:946–52. doi: 10.1097/ACM.0b013e3182222887, PMID: 21694564

[26] Pincus HA Henderson B Blackwood D Dial T. Trends in research in two general psychiatric journals in 1969-1990: research on research. Am J Psychiatry. (1993) 150:135–42. PMID: 8417556 10.1176/ajp.150.1.135

[27] Bignami G De Girolamo G Fava GA Gaston A Morosini PL Pasquini P. The impact on the international literature of the scientific production of Italian researchers in the disciplines ‘psychiatry’ and ‘psychology’. A bibliometric evaluation. Epidemiol Psichiatr Soc. (2000) 9:11–25. doi: 10.1017/S1121189X00007715, PMID: 10859872

[28] Süßenbacher S Amering M Gmeiner A Schrank B. Gender-gaps and glass ceilings: a survey of gender-specific publication trends in psychiatry between 1994 and 2014. Eur Psychiatry. (2017) 44:90–5. doi: 10.1016/j.eurpsy.2017.03.008, PMID: 28550785

[29] Saraykar S Saleh A Selek S. The association between NIMH funding and h-index in psychiatry. Acad Psychiatry. (2017) 41:455–9. doi: 10.1007/s40596-016-0654-4, PMID: 28063125

[30] Bornmann L Mutz R Daniel HD. Gender differences in grant peer review: a meta-analysis. J Informet. (2007) 1:226–38. doi: 10.1016/j.joi.2007.03.001

[31] Hafeez DM Waqas A Majeed S Naveed S Afzal KI Aftab Z. Gender distribution in psychiatry journals’ editorial boards worldwide. Compr Psychiatry. (2019) 94:152119. doi: 10.1016/j.comppsych.2019.152119, PMID: 31473553

[32] González-Alvarez J Cervera-Crespo T. Psychiatry research and gender diversity: authors, editors, and peer reviewers. Lancet Psychiatry. (2019) 6:200–1. doi: 10.1016/S2215-0366(19)30039-2, PMID: 30744998

[33] Kecmanovic D Hadzi-Pavlovic D. Psychiatric journals as the mirror of the dominant psychiatric model. Psychiatrist. (2010) 34:172–6. doi: 10.1192/pb.bp.108.024018

[34] Silver JK. Gender equity on journal editorial boards. Lancet. (2019) 393:2037–8. doi: 10.1016/S0140-6736(19)31042-6 31106748

[35] Angermeyer MC Winkler I. Who, what, how much, where? - an analysis of publications by German authors on sociopsychiatric issues in scientific journals. Psychiatr Prax. (2001) 28:368–75. doi: 10.1055/s-2001-18618, PMID: 11721222

[36] Sharifi V Rahimi-Movaghar A Mohammadi MR Goodarzi RR Izadian ES Farhoudian A. Analysis of mental health research in the Islamic Republic of Iran over 3 decades: a scientometric study. East Mediterr Health J. (2008) 14:1060–9. PMID: 19161078

[37] Zhang J Chen X Gao X Yang H Zhen Z Li Q. Worldwide research productivity in the field of psychiatry. Int J Ment Heal Syst. (2017) 11:1–6. doi: 10.1186/s13033-017-0127-5 PMC5310092 28289438

