# Supplementary material for: Female researchers in high-impact psychiatric journals: What do they focus on?
Source: Front Psychiatry. 2023 Feb 14;14:1104683. doi: 10.3389/fpsyt.2023.1104683 (PMC9973523; doi:10.3389/fpsyt.2023.1104683)
Supplement: Supplementary file 1 [file Data_Sheet_1.pdf]

Supplementary Material

Female researchers in high-impact psychiatric journals: What do they focus on?

Melanie Trimmel[1], Michaela Amering[1], Stefanie Suessenbacher-Kessler[1], Beate Schrank[2], Andrea Gmeiner[1*]

1 Clinical Division of Social Psychiatry, Department of Psychiatry and Psychotherapy, Medical University of Vienna, Vienna, Austria

2 Department of Adult Psychiatry, Karl Landsteiner University for Health Sciences, University Clinic Tulln, Tulln, Austria

*** Correspondence:**

Corresponding Author: Andrea Gmeiner, MD

andrea.gmeiner@meduniwien.ac.at

1 Supplementary Data

The classification of subspecialty areas is described below.

(1) general mental health (2) forensic (3) addiction (4) consultation-liaison psychiatry (5) cross-cultural (6) ethics and human rights, philosophy, concepts (7) policy (8) gender issues (9) woman's (health) issues (10) men's (health) issues (11) neuroimaging (12) genetics (13) methodology (14) physical therapies (electroconvulsive therapy, magnetic stimulation, neurosurgery) (15) patients defined from other medical field (16) somatic illness in psychiatric patients (17) endocrinology (17) post mortem brain study (18) animal study (19) research on research (20) gay issues (23) informal caregivers, relatives as caregivers (24) behaviour issues, challenging behaviour, violence, aggression (25) other (26) aetiology (27) psychotherapy and psychological intervention (28) pharmacotherapy (29) gerontopsychiatry (30) database (31) prevention (32) cognition (33) stigma (34) self-harm (35) psychopathology, diagnostics, nomenclature (36) army (37) gene-environment-interaction (38) biomarkers (39) primary care
